# Financial burden for caregivers of adolescents and young adults with cancer

**DOI:** 10.1002/pon.5937

**Published:** 2022-04-20

**Authors:** Chandylen L. Nightingale, Mollie R. Canzona, Suzanne C. Danhauer, Bryce B. Reeve, Dianna S. Howard, Reginald D. Tucker-Seeley, Shannon L. S. Golden, Denisha Little-Greene, Michael E. Roth, David E. Victorson, John M. Salsman

**Affiliations:** 1 Department of Social Sciences & Health Policy, Wake Forest School of Medicine, Winston Salem, NC, USA; 2 Department of Communication, Wake Forest University, Winston Salem, NC, USA; 3 Department of Population Health Sciences, Duke University School of Medicine, Durham, NC, USA; 4 Department of Internal Medicine, Section on Hematology and Oncology, Wake Forest School of Medicine, Winston Salem, NC, USA; 5 Leonard Davis School of Gerontology, University of Southern California, Los Angeles, CA, USA; 6 Goldsmith Research Group, Winston Salem, NC, USA; 7 Department of Pediatrics, MD Anderson Cancer Center, Houston, TX, USA; 8 Department of Medical Social Sciences, Northwestern University Feinberg School of Medicine, Chicago, IL, USA

**Keywords:** adolescent, adolescent and young adult oncology, cancer, caregiver, financial burden, financial distress, financial toxicity, oncology, psycho-oncology, young adult

## Abstract

**Objective:**

Adolescent and young adult (AYA) cancer survivors are vulnerable to cancer-related financial burden, which is likely shared by their caregivers. This study aims to enhance an existing conceptual model of financial burden by conducting concept elicitation interviews with caregivers to generate knowledge that can be translated to inform instrumental and psychosocial support in cancer care.

**Methods:**

Qualitative concept elicitation interviews were conducted with 24 caregivers of AYA cancer survivors (caregivers of adolescents, *n* = 12; caregivers of emerging adults, *n* = 12) recruited from four sites. Constant comparative methods were used to identify themes, and results were interpreted and organized into domains of the conceptual model. We also explored COVID-19 related financial impacts among a subset (*n* = 12) of caregivers.

**Results:**

Seven themes emerged, which varied by age group and strengthened the conceptualization of the model. Themes centered on: (1) direct and indirect costs of cancer; (2) impact of socioeconomic status on financial burden; (3) caregiver desire to shield AYAs from distress due to financial burden; (4) strategies to manage cancer-related costs; (5) worries about AYAs’ financial future; (6) seeking and receiving financial support; and (7) navigating the healthcare system. Findings also revealed that COVID-19 exacerbates financial burden for some caregivers.

**Conclusions:**

Building upon our prior work, we have adapted the conceptual model of financial burden to reflect perspectives of AYAs, oncology providers, and now, caregivers. An important next step is to develop a reliable and valid self-report measure of financial burden among caregivers of AYA cancer survivors.

## 1 | BACKGROUND

Informal (unpaid/family) cancer caregivers are essential members of the health care team, providing an average of 33 h a week caring for their loved one.^1^ Though caring for a loved one with cancer can be rewarding, the negative implications for the caregiver can be broad and substantial.^2–4^ A scant, but growing body of literature describes cancer-related financial burden among informal (unpaid/family) caregivers with 25% of cancer caregivers reporting high levels of financial burden, often secondary to interruptions in employment (e.g., taking time off, taking a leave of absence) and costs associated with cancer care.^1,5–7^ External financial resources for cancer caregivers are limited, and caregivers report high unmet financial needs.^5, 8^ Increased financial burden in caregivers negatively impacts patients’ health and care delivery as it is associated with poorer treatment adherence and quality of life.^9^

Cancer-related financial burden may be exacerbated in those providing care to adolescent and young adult (AYA) survivors (i.e., those diagnosed with cancer from the ages of 15–39 years), a population at high risk for having inadequate insurance coverage and limited financial assets.^10–13^ Compared to other age groups with cancer, AYAs self-report greater financial burden.^10–12^ Caregivers often share or shoulder the responsibility for their loved one’s cancer-related costs, and this burden may be greater and more persistent among those caring for younger AYAs (ages 15–25 years), as this group is beginning to navigate financial independence.^5,14^ In addition to caregivers contributing to treatment-related costs, caregivers of young adults may also need to abruptly take responsibility for the young adult’s living expenses, as they may be more likely to move in with their caregiver during treatment.^15^ Further, unlike adult cancer patients who have an active role in managing the costs associated with their cancer care, adolescent cancer survivors report limited awareness of costs, which are instead managed by a family caregiver.^16^

**FIGURE 1:**
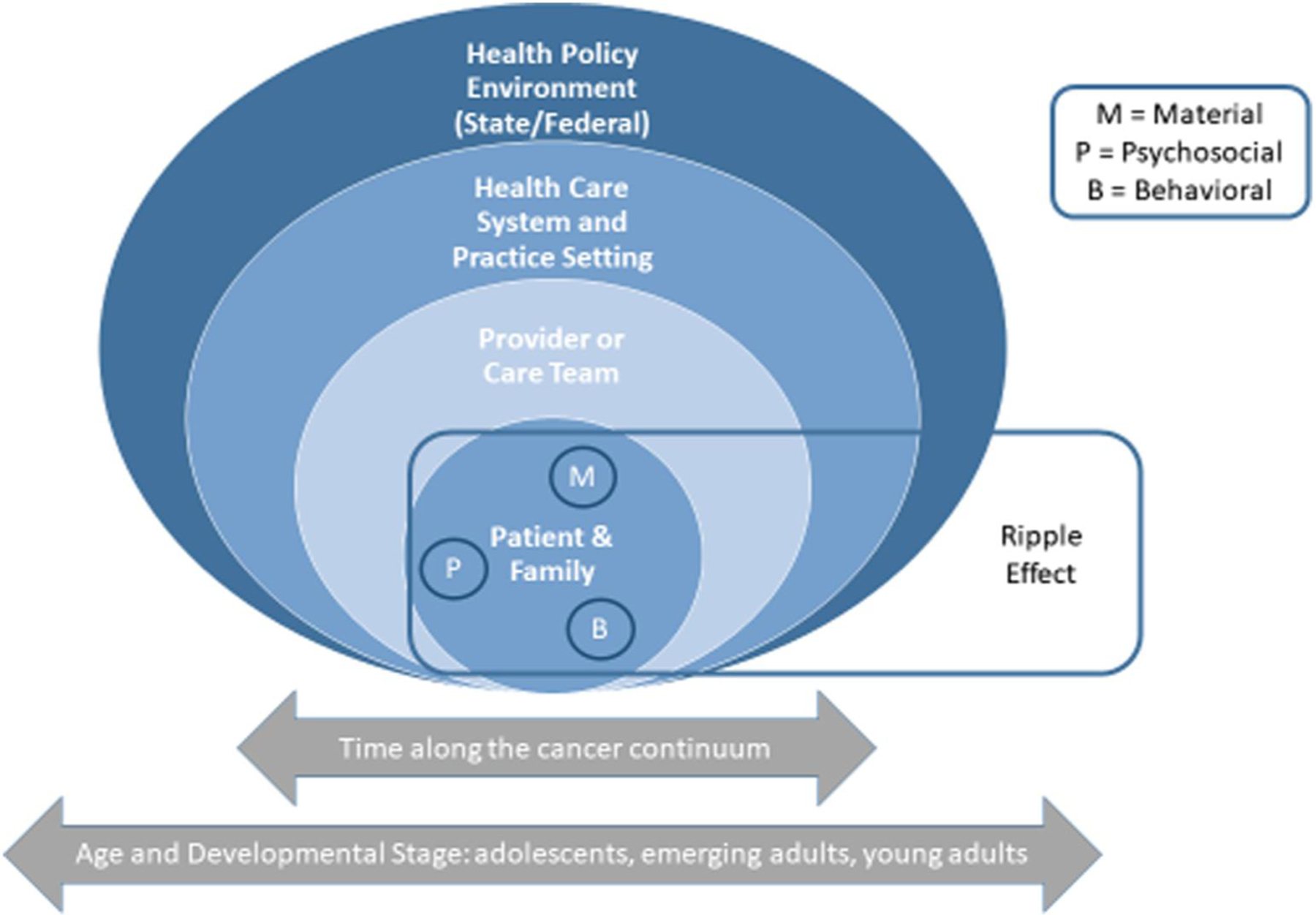
Adapted conceptual model of financial burden^19,20^

To our knowledge, no qualitative studies have explored the financial burden of cancer on caregivers of AYAs, though studies have documented substantial financial burden among parents of children with cancer.^17–19^ Our team recently conducted a systematic review and found financial burden measures are sparse for cancer caregivers and non-existent for caregivers of AYAs.^20^ A critical first step to understanding the multidimensional financial impact of cancer on caregivers of AYAs is to develop a psychometrically sound measure that specifically captures cancer-related financial burden among caregivers. Accordingly, we conducted qualitative concept elicitation interviews with stakeholders to refine a conceptual model for financial burden of cancer in AYAs as part of a standardized measurement approach (See Figure 1).^16,21^ We previously reported on the conceptual model following AYA survivor and provider interviews, which reflects the multiple layers of impact and influence on financial burden including AYA patients/families, health care providers, the broader healthcare system, and the health policy environment.^16^ Further, the model depicts critical interrelated material, psychosocial, and behavioral aspects of experience that characterize financial burden for patients and families and the “ripple effects” of indirect costs related to cancer.^16^ This study aims to add to the existing model by capturing salient domains of financial burden focusing on the caregiver perspective to enhance instrumental and psychosocial support in cancer care. In this paper, we: (1) perform data source triangulation by presenting findings on caregivers’ financial burden and how it relates to the conceptual model^22^; (2) explore how financial burden varies for caregivers of adolescents (15–17 years old) and emerging adults (18–25 years old); and (3) explore financial burden associated with COVID-19 during or after cancer treatment.

## 2 | METHODS

### 2.1 | Study design

Semi-structured qualitative concept elicitation interviews were conducted with 24 caregivers of AYAs between 13 November 2018 and 12 March 2021.

### 2.2 | Recruitment

Caregivers of AYA cancer survivors were eligible if they: (1) were ≥18 years of age; (2) were able to read and understand English; (3) had a loved one for whom they were providing care and at least partly financially responsible; and (4) had a loved one who met criteria as an eligible AYA cancer survivor participant in the larger study.^16^ Participants were recruited from the Wake Forest Baptist Comprehensive Cancer Center (WFBCCC), the Robert H. Lurie Comprehensive Cancer Center of Northwestern University (RHLCCC), Children’s Healthcare of Atlanta, and MD Anderson Cancer Center. Caregivers were approached in clinic or contacted by phone after first screening the care-recipient’s electronic health record to determine AYA eligibility, per the larger study.^16^ This study was approved by the local institutional review boards at WFBCCC and RHLCCC who were responsible for consenting participants (IRB00044525 at WFBCCC; STU00208207 at RHLCCC). Caregivers provided written informed consent and received a $25 gift card and $25 donation to a cancer support agency of their choice.

### 2.3 | Data collection

Semi-structured interviews (Mean = 46 min) were conducted with 24 caregivers of AYA patients (adolescents, *n* = 12; emerging adults, *n* = 12). Interviews were conducted in-person (*n* = 8; WFBCCC only), by video conference (*n* = 12), or by phone (*n* = 4). All interviews were conducted through the Qualitative and Patient Reported Outcomes (Q-PRO) Shared Resource at the WFBCCC by a trained research associate who has expertise in qualitative methods. The interview guide followed a funnel approach with open-ended questions eliciting participants’ understanding and experience of financial aspects of cancer with subsequent follow-up questions exploring material (e.g., financial resources and deficits), psychosocial (e.g., distress related to financial hardship), and behavioral (e.g., cost-cutting strategies) factors that shape and/or characterize their perception of financial burden.

Data collection was halted briefly in the spring of 2020 due to COVID-19 restrictions. During that time, the study team revised the interview guide to include questions specific to the financial burden of COVID-19. Therefore Wave 1 (*n* = 12) did not include COVID-19-related discussion but Wave 2 (*n* = 12) did. All interviews were recorded.

### 2.4 | Data analysis

All interviews were outsourced for transcription. Each transcript was reviewed by the interviewer for accuracy. Two Q-PRO research associates reviewed all transcripts to devise and test conceptual codes. Transcripts were coded in tandem; coding discrepancies were resolved with discussion. Final primary analyses were stratified by participant group (caregivers of adolescents; caregivers of emerging adults). Data saturation was evaluated at the theme level within each strata. Interviews within each strata were divided into sets of four based on chronological order. Themes were considered saturated if they were present in at least one interview within each set.^23^ We reviewed the thematic categories and associated data to identify if and how themes fit within domains previously identified in the conceptual model. Domain affiliations were discussed and confirmed by study authors. Differences were resolved by consensus.^16^ Data were stored and managed using ATLAS. ti (Version 7.5).

## 3 | RESULTS

Caregivers were a parent (*n* = 23) or grandparent custodian (*n* = 1) to an AYA (caregiver characteristics are presented in Table 1). Seven themes emerged characterizing caregivers’ financial burden. The following section: (1) presents themes with associated properties and how they mapped onto the material conditions (direct and indirect costs, productivity loss, medical debt/bankruptcy), psychosocial experiences (stress, distress, and worry about medical costs or concerns about work impairment associated with cancer), and behavioral actions/strategies (coping behaviors and strategies such as delaying or forgoing medical care, cutting costs) domains of the conceptual model (Figure 1)^16^; (2) identifies theme variation by age group; and (3) summarizes COVID-19-related financial impacts. All themes mapped onto at least one domain of the conceptual model. Supplemental Table 2 provides a summary of caregiver financial burden themes and related conceptual model domains.^16^ See Supplemental Table 3 for themes, theme properties, exemplar quotes, and theme variation by age group.

### 3.1 | Theme 1: Socioeconomic status at the time of diagnosis and treatment can amplify or mitigate financial burden

Caregivers reported financial burden was mitigated by: 1) steady income, especially multiple stable salaries from two-income house-holds; and, 2) health insurance (public, private, Medicaid) that secured the ability to pay for direct and indirect costs of cancer. Those who reported unemployment or non-salaried positions with inconsistent work schedules (e.g., contract work) described distress stemming from fear of unpredictable future cancer-related expenses. This theme mapped onto all three domains of the conceptual model.

### 3.2 | Theme 2: Caregivers’ financial burden includes direct and indirect costs related to cancer/cancer care

Caregivers discussed the impact of direct and indirect costs of cancer. Caregivers cited difficulty and distress related to paying insurance premiums and copays for treatments, medical appointments, procedures, and prescriptions. Orthopedic equipment, wigs, particular foods (due to nausea), and additional cleaning supplies were needed to manage cancer treatments. Participants discussed significant travel and relocation costs. Gasoline was needed for traveling to treatment centers, in addition to parking, food, and childcare costs associated with travel. Relocating and maintaining two properties and/or mortgages was also discussed specifically for families whose children required bone marrow transplants or radiation treatments. This theme mapped onto all three domains of the conceptual model.

### 3.3 | Theme 3: Caregivers shield AYAs from financial burden

Caregivers consistently described the belief that financial burden is a stressor an AYA with cancer “does not need.” They fear AYAs will become “overwhelmed,” “worried,” “anxious,” or “crushed.” Consequently, caregivers employed strategies to “shield” or “shelter” AYAs. Caregivers reported hiding medical bills from AYAs, omitting information about the extent of the impact on the caregiver’s finances, minimizing depth and specificity of information disclosed (e.g., “we’re in a tight spot”), or admitting financial challenges but assuring AYAs they can be managed. This theme mapped onto all three domains of the conceptual model.

### 3.4 | Theme 4: Caregivers seek and/or receive financial support from social networks and healthcare institutions/organizations

Caregivers sought and received financial support from organizations, family and friends, and community (school, employers, Church) event-based, in-person, and social media fundraisers. Insurance companies, hospitals, and cancer centers also assigned case workers/navigators who connected caregivers with financial resources for treatment costs, lodging, travel expenses, and food. This theme mapped onto all three domains of the conceptual model.

### 3.5 | Theme 5: Caregivers desire help navigating the healthcare system and locating financial resources

Caregivers reported they would like assistance navigating financial aspects of the healthcare system and locating financial resources. They described receiving information about procedures, costs, and internal/external resources in “bits and pieces” at “random times” via intermittent phone calls from different people across the cancer trajectory. They described a desire for face-to-face meetings with one dedicated financial case worker/navigator, clear written documentation of costs and resource contact information, and email summaries of meetings. At times, financial assistance/resources were delivered after significant debt had accrued. They reported they would like assistance managing deadlines to ensure payments are made on schedule. This theme mapped onto all three domains of the conceptual model.

### 3.6 | Theme 6: Caregivers use strategies to prepare for and adapt to cancer-related costs

Caregivers suggested they would do whatever is needed to pay cancer-related costs and “resolve” financial consequences; they employed multiple strategies to prepare for/adapt to those costs. Caregivers reported delaying unnecessary travel and vacations and reducing non-essential expenses for themselves and their children. They took steps to maximize their financial resources including applying for loans, deferring existing debt payments, using savings, working higher-paying (and often less-enjoyable) jobs, longer hours, or multiple jobs. They reported researching cancer treatments, hospital procedures, and insurance systems to plan ahead financially. They delayed their own medical appointments or delayed filling AYA prescriptions to wait for generic drugs or meet deductibles. Caregivers reported that managing the AYA’s cancer prompted them to reevaluate their spending philosophy as a parent/caregiver and renewed their intent to save money. This theme mapped onto the psychosocial and behavioral domains of the conceptual model.

### 3.7 | Theme 7: Caregivers worry about AYAs’ medical and financial futures

Caregivers discussed concerns about future financial burden. They reported worry and uncertainty about future expenses, cancer-related sequelae, or other cancer or non-cancer-related health problems and whether they/their children would be prepared financially. For those earlier in the cancer trajectory, treatment options and insurance coverage were still sources of uncertainty. Given AYAs’ ages, their career trajectories and financial futures were also still in question. This theme mapped onto the psychosocial domain of the conceptual model.

### 3.8 | Thematic age group comparisons

Themes emerged in varied and nuanced ways by AYA age group. Both groups reported *financial burden includes direct and indirect costs related to cancer/cancer care (Theme 2)*. Some emerging adults were able to assist caregivers with costs. Caregivers of adolescents were often solely financially responsible. Caregivers of adolescents and emerging adults suggested *socioeconomic status* (including insurance coverage) *at diagnosis and treatment can amplify or mitigate financial burden (Theme 1)*. Caregivers of adolescents reported cancer-related expenses were minimal because of Medicaid (e.g., Medicaid does not require co-pays, etc.) or because they were covered on a parent’s insurance plan. Emerging adults approaching 26 years of age would soon no longer be age-eligible for coverage under a parent’s insurance. Both groups of *caregivers use strategies to prepare for and adapt to cancer-related costs (Theme 6)*. Caregivers of adolescents reported the need to adjust spending that impacted other children in the family (extracurricular classes, school activities, etc.). Both groups of *caregivers shield AYAs from financial burden (Theme 3)*. However, caregivers of adolescents more consistently and forcefully asserted it was their sole responsibility to think about and manage expenses. They *worried about AYAs’ medical and financial futures (Theme 7)* because adolescents had not begun to make career decisions. Caregivers of adolescents also reported concern that cancer would delay or interfere with developmental milestones that could impact their future finances.

### 3.9 | COVID-19-related financial impacts

Analyses revealed the COVID-19 pandemic affected financial burden (see Supplemental Table 4). While some caregivers’ income remained consistent (e.g., essential workers) or increased (suppliers of critical materials or resources), many caregivers, families, and AYAs experienced *job loss or reduction inwork hours*. Financial distress was magnified by the *uncertain nature of the pandemic and its long-term impact on the job market*. Caregivers experienced this loss/reduction in income and uncertainty at the same time they experienced an *increase in household size*. These family “bubbles” stemmed from loved ones’ inability to pay rents/mortgages or from children returning from college when universities moved to remote learning. Additional people in these households reported increased food and utility expenses. Caregivers struggled as the *cost of goods and services increased*. Managing *cost of purchasing pandemic-related products, masks, and cleaning supplie*s was challenging. Caregivers reported difficulty maintaining employment due to *reduced home-based caregiving and support*. Difficulty *finding and affording medications due to limited inventory* was also reported.

## 4 | DISCUSSION

This study is the first to explore experiences of financial burden among caregivers of AYA survivors. These findings deepen our understanding of material, psychosocial, and behavioral domains of the adapted patient- and provider-informed conceptual model of cancer-related financial burden, in particular, illuminating nuanced experiences of people caring for cancer patients in different phases of the AYA lifespan.^16,21^ Further, this study uncovered insights about financial burden and AYA cancer caregiving, including the impact of the evolving COVID-19 pandemic, which can be used to enhance instrumental and psychosocial support in cancer care and the timing of that support.

### 4.1 | Conceptual model of financial burden and lifespan developmental insights

Results reinforce and extend the original material-psychosocial-behavioral conceptual model that we adapted based on AYA patient and provider perspectives.^16,21^ Insurance coverage and financial assistance (material domain) shaped participants’ psychosocial and behavioral experiences in both studies. In our prior work, emerging adult AYA participants reported the ability to assist with cancer costs, like traveling to treatment centers and paying medical bills, but they also reported varied, less consistent sources of insurance.^16^ In this study, caregivers of emerging adults who were approaching 26 years of age discussed concerns about future insurance coverage. They suggested emerging adults may not be age-eligible for future coverage through caregiver’s insurance, which magnifies financial burden. Further, we previously found though adolescents and older AYAs report financial burden, adolescents described extensive financial assistance through social networks and special assistance programs as compared to older AYAs.^16^ Providers in our prior study posited that young patients receive more assistance than older AYAs because people are more motivated to donate to younger cancer patients.^16^ In addition, adolescents are often treated in pediatric centers where there are more resources to support patients and their families. Thus, caregivers of emerging adults being treated in adult oncology settings without AYA-specific resources may face this unique financial challenge related to age.^15^

A communication dynamic involving AYA knowledge of financial circumstances (material domain) and associated psychosocial and behavioral experiences for AYAs and caregivers emerged. In our prior work, adolescents expressed less awareness and depth of understanding regarding financial aspects of cancer and financial distress.^16^ Caregivers in this study, particularly caregivers of adolescents, reported the unwavering belief that AYAs should not be burdened with this knowledge and used strategies to shield AYAs from financial concerns, including strategies that may be harmful to the caregivers’ psychosocial and physical health (such as delaying caregiver medical care). Shouldering the responsibility to protect adolescents from the financial realities of cancer is a particularly salient aspect of these caregivers’ experiences and is consistent with parental caregiver experiences of children with cancer.^24^

In our prior work, younger patients focused on material conditions related to cancer costs and implications for their social lives.^16^ AYA patients in that study described how financial issues restricted spending on social activities, resulting in isolation. Caregivers in this study reported concern about limiting expenditures on extracurricular activities, but also reported concerns regarding AYAs’ uncertain medical and financial futures. Thus, caregivers are tasked with continually managing the dialectic between making financial choices that allow AYAs to engage socially while protecting their future financial health.

### 4.2 | Clinical implications

This study highlights the need for healthcare professionals to be aware that age, employment status, and level of caregiver support have critical implications for financial burden. Younger AYAs often receive financial support from caregivers, family, and community resources (e.g.*, friends, schools, employers, religious communities)*, and special assistance programs. Younger AYAs and their caregivers who don’t have a strong broad support community may not be aware of or have access to this kind of financial assistance. Providers can enhance their awareness of these issues by using patient-centered communication strategies. Patient-centered communication focuses on helping providers understand a patient’s biopsychosocial context, fostering trusting relationships, and facilitating the information exchange necessary for delivering multidimensional care.^25^ Asking open-ended questions about a patient family and social context and exploring how and if these factors impact their cancer care experience can help providers understand AYA/caregiver financial information and support needs.

Healthcare system policies regarding age and features of AYAs’ developmental stage exacerbate the impact of financial burden. While emerging adults benefit from expanded eligibility under parental health insurance coverage under the Affordable Care Act, as referenced above, they may turn 26 during their cancer care and fall into a gap where they are too old to qualify for insurance coverage through caregivers, may not be eligible for Medicaid, and/or still be in the process of establishing their professional and financial lives, obstructing the ability to access or afford employer-based coverage and creating an additional source of uncertainty.

Caregivers are tasked with managing numerous sources of financial uncertainty: what does treatment cost (event uncertainty), can we afford it and could it impact their outcomes (efficacy and outcome uncertainty) and what will AYAs’ financial future look like (temporal uncertainty). Navigating multiple uncertainties compounds the associated stress, which can negatively impact caregiver and AYA psychosocial, social/relational, and physical health outcomes.^26^ At the same time, caregivers navigate this uncertainty, they often reported attempting to protect AYAs by continually omitting or hiding financial burden information. Concealing information during times of stress has been linked to rumination and may magnify caregivers’ psychological distress.^27^ Eliciting information about financial dimensions of families’ cancer experience can help inform appropriate referrals to counseling services and psychosocial oncology providers.

Navigating complex insurance coverage interruptions is challenging during this already stressful time. Caregivers described general difficulty understanding the financial landscape of cancer care and finding/accessing resources. Information was incomplete, unclear, delivered sporadically, and late. Financial navigation interventions have primarily been patient-targeted, with minimal inclusion of the caregiver.^28–30^ Our results suggest a formal process including regular meetings with a financial resource navigator at diagnosis and across the cancer continuum is warranted for caregivers of AYAs. Written summaries from these meetings with next steps and timelines would provide caregivers with a more effective “map” of expectations for the financial terrain. Encouraging notetaking and allowing patients to audio record interactions can aid with recall when written summaries are not feasible.

Caregivers would benefit from financial planning resources to prepare for and adapt to cancer-related costs. Similar to other studies, caregivers described coping strategies (e.g., working multiple stressful jobs) that could have negative health consequences for caregivers, AYAs and their families.^5,31^ Helping caregivers arrive at a more holistic understanding of their financial circumstances and assess options for mitigating financial burden could prevent cancer-related distress. A recent study of practice settings within the National Cancer Institute (NCI) Community Oncology Research Program suggests the use of the National Comprehensive Cancer Network (NCCN) distress thermometer problem checklist as part of standard distress screening may be helpful in detecting financial burden across the cancer continuum. Further, community practice settings with a cancer-specific financial navigator were more likely to assess financial burden and identify areas of intervention for individual practices and patient groups.^28^

If navigation services are not regularly accessible, then providers, nurses, and other healthcare practitioner or administrators may initiate discussions and connect patients to resources such as The NCI Cancer Information Service telephone helpline.^32,33^ Patients can also be referred to searchable websites/organizations such as Triage Cancer, Samfund Grants, and Cancer and Careers for helpful information about local and national resources.^32^

Additionally, our findings and others suggest providers and health care professionals need to be aware of potential constraints imposed by the COVID-19 pandemic (e.g., change in employment status, difficulty maintaining employment, increased household size, etc.) and be prepared to assess how the pandemic intersects with AYA cancer patients’ and families’ financial health through the use of the patient-centered communication strategies discussed.^34^ These caregivers require informational, instrumental, and psychosocial support to manage evolving financial tensions.

The feasibility of different approaches must be assessed by healthcare practice. Size, resources available, and vulnerability of patient groups vary by cancer care setting. There is no “gold standard” for recommended screening tools and financial resources as pathways are often fragmented and ill defined.^28^ Healthcare practices should consider the degree to which current procedures and patient level factors affect financial burden for AYAs and adopt actionable strategies such as supporting patient-provider financial communication, training existing employees in financial navigation, partnering with community agencies, creating/gathering and disseminating informational resources.

### 4.3 | Study limitations

This study is limited by our sample. The majority of caregivers were non-Hispanic White, female, with at least some college education, an annual income of ≥ $60,000, and providing care for an AYA with private health insurance. These factors may attenuate financial hardship among caregivers and potentially limit the generalizability of our findings. Further, all caregivers were a parent or grandparent of an AYA and did not include spouses or intimate partners of emerging adults; however, we believe that given the age of younger AYAs (15–25 years of age), caregivers are more likely to be a parent/grandparent, potentially making our sample reflective of the broader caregiver population for younger AYAs. Finally, participants were caring for AYAs who were currently receiving active cancer treatment or were up to 5 years post-treatment. As such, we were unable to explore long-term financial burden among caregivers.

### 4.4 | Strengths

This study is strengthened by the rigorous qualitative methods that were used to collect and analyze data.^23^ In addition, we compared our findings for caregivers of adolescents and caregivers of emerging adults and found important differences for caregivers of these two age groups. Further, while studies have been conducted that highlight the validity of the material-psychosocial-behavioral conceptual model of cancer-related financial burden, we have adapted this model to reflect perspectives of AYA cancer survivors, oncology providers, and informal caregivers of AYAs.^16,21,35–37^

### 4.5 | Future directions

This study is part of a larger initiative to develop self-report measures of financial burden for AYAs and their caregivers and builds upon our team’s recent systematic review of financial burden measures and concept elicitation interviews with AYA cancer survivors and oncology providers.^16,20^ These collective findings have guided the development of a comprehensive set of items (questions) that assess financial burden. Next steps will focus on conducting cognitive interviews to evaluate the comprehensibility of our preliminary item pools for the financial burden measures. This rich, qualitative data has yielded an in-depth understanding of the experiences of financial burden and will inform measures that reflect the experiences of AYAs and their caregivers. Future research should explore potential differences in financial burden for parental versus intimate partner caregivers and long-term financial burden among AYAs and their loved ones. Improved measurement can guide the development and testing of interventions to minimize the impact of financial burden on AYA cancer survivors and their informal caregivers.

## Supplementary Material

Supplement 1 Financialburdenforcaregiversofadolescentsandyoungadultswithcancer

Supplement 2 - Financialburdenforcaregiversofadolescentsandyoungadultswithcancer

Supplement 3 - Financialburdenforcaregiversofadolescentsandyoungadultswithcancer

## Figures and Tables

**TABLE 1:** Sample characteristics (*n* = 24)

Category			Adolescents	Emerging adults
**Age**	49.1 (*M*)	7.0 *(SD)*		
	**N**	**%**	**N**	**N**
**Relationship to an AYA with cancer**				
Mother			10	10
Father			1	2
Grandparent			1	0
**Sex**				
Female	20	83.3	10	10
Male	4	16.7	2	2
**Ethnicity**				
Hispanic origin	4	16.7	3	1
**Race** ^a^				
White	18	75.0	9	9
Black/African American	4	16.7	1	3
Other	1	4.2	1	0
**Education**				
Some high school	1	4.2	1	0
High school grad/General Education Diploma	3	12.5	2	1
Some college/Technical degree/Associate of Arts	9	37.5	4	5
College degree (Bachelor of Arts/Bachelor of Science)	1	4.2	1	0
Graduate school	10	41.7	4	6
**Relationship status**				
Married/Living with partner in committed relationship	17	70.8	7	10
Never married	2	8.3	2	0
Divorced/Separated	5	20.8	3	2
**Primary income earner**				
Self	10	41.7	3	7
Spouse/Significant other	8	33.3	6	2
Self & spouse/Significant other contribute equally	4	16.7	2	2
Child	1	4.2	0	1
Parents	1	4.2	1	0
**Primary health insurance**				
Private insurance (employer-provided)	19	79.2	10	9
Medicare	3	12.5	1	2
Medicaid	1	4.2	1	0
Veterans/Military insurance	1	4.2	0	1
**Annual household income**				
Less than $15,000	3	12.5	2	1
$15,000 to $29,999	2	8.3	1	1
$30,000 to $59,999	3	12.5	2	1
$60,000 to $100,000	3	12.5	2	1
More than $100,000	13	54.2	5	8
**Financial dependents**				
None	3	12.5	1	2
1–2	11	45.8	6	5
3–4	7	29.2	4	3
5 or more	3	12.5	1	2
**Pay for costs associated with child/partner’s cancer care?**				
No, I do not pay for any of these costs	2	8.3	1	1
Yes, I pay for some of these costs	9	37.5	5	4
Yes, I pay for most of these costs	4	16.7	1	3
Yes, I pay for all of these costs	9	37.5	5	4
**Caregiver’s child’s primary health insurance**				
Private insurance (employer-provided)	14	58.3	8	6
Medicare	3	12.5	2	1
Medicaid	3	12.5	2	1
S/he doesn’t have any health insurance	2	8.3	0	2
Private insurance (purchased directly)	1	4.2	0	1
Veterans/Military insurance	1	4.2	0	1
**Hours spent providing care**				
Less than 1 h a day	10	41.7	5	5
1–2 h a day	3	12.5	1	2
3–4 h a day	5	20.8	3	2
More than 8 h a day	6	25.0	3	3
**Provides unpaid care to an additional child/adults with special healthcare needs**				
Yes	2	8.3	0	2
No	22	91.7	12	10

a Missing, *n* = 1(4.2%).

## Data Availability

The data that support the findings of this study are available from the corresponding author upon reasonable request.
