# Supplementary material for: Financial burden for caregivers of adolescents and young adults with cancer
Source: Psychooncology. Author manuscript; Available in PMC 2023 Aug 1. (PMC9540021; doi:10.1002/pon.5937)
Supplement: Supplement 1 Financialburdenforcaregiversofadolescentsandyoungadultswithcancer [file NIHMS1831818-supplement-Supplement_1_Financialburdenforcaregiversofadolescentsandyoungadultswithcancer.docx]

**Table 2.** Summary of Themes for Caregiver Financial Burden by Conceptual Framework Dimension^19-20^

| **Themes** | **Dimensions of Conceptual Model** | | |
| --- | --- | --- | --- |
|  | **Material** | **Psychosocial** | **Behavioral** |
| Socioeconomic status at the time of diagnosis and during treatment can amplify or mitigate financial burden | x | x | x |
| Caregivers’ financial burden includes direct and indirect costs related to cancer/cancer care | x | x | x |
| Caregivers shield AYAs from financial burden | x | x | x |
| Caregivers seek and/or receive financial support from social networks and health care institutions/organizations | x | x | x |
| Caregivers desire help navigating the healthcare system and finding resources | x | x | x |
| Caregivers use strategies to prepare for and adapt to cancer-related costs |  | x | x |
| Caregivers worry about AYAs’ uncertain medical and financial future |  | x |  |
