# Supplementary material for: Financial burden for caregivers of adolescents and young adults with cancer
Source: Psychooncology. Author manuscript; Available in PMC 2023 Aug 1. (PMC9540021; doi:10.1002/pon.5937)
Supplement: Supplement 2 - Financialburdenforcaregiversofadolescentsandyoungadultswithcancer [file NIHMS1831818-supplement-Supplement_2_-_Financialburdenforcaregiversofadolescentsandyoungadultswithcancer.docx]

**Table 3.** Themes, Theme Properties, Exemplar Quotes, and Theme Variation by Age Group

| **Theme** | **Properties** | **Exemplar Quotes** | **Theme Variation by Age Group** |
| --- | --- | --- | --- |
| Socioeconomic status at the time of diagnosis and during treatment can amplify or mitigate financial burden | - Unemployment/job loss - Inconsistent/reduced work schedules - Steady income - Two house-hold income   Health insurance coverage | “. . . as the months went by, and then I was working less, it started becoming more of a challenge because there's only so much saving that you have, it won't last you long. Then, you're away from home (relocated). You're not working the way you were working before. My husband was out, so his paycheck was cut, as well.” (21203)  “I would do a lot of contract work prior to him being diagnosed . . . I am not able to do that anymore.” (11201)  “We were fortunate enough that we were both able to work during her treatment time, and so we still had our salaries as normal during that time . . . If one of us had had to not work, that would have been a different scenario.” (11203)  “Most all of her costs were covered under Medicaid. So, we didn't have to worry with copays . . .” (11205)  “She's on mine (plan). And it's a good insurance. We’re very fortunate.” (11209) |  |
| Caregivers’ financial burden includes direct and indirect costs related to cancer/cancer care | - Insurance premiums, copays for treatments, procedures, medical appointments, and prescriptions - Orthopedic equipment, wigs, special foods, additional cleaning supplies - Travel, relocation, and childcare costs | “I had a $5000 deductible and then a 20% copay after that, so the medical bills for someone in my situation are crushing because I don't have the resources to pay that. After that it did kick in and pay 100% and I'm grateful for that…. I am still paying, and I'll be paying, the hospital a long time because for some people $5000 out-of-pocket and 20% maybe is not such a big deal, but for me in our situation it's a pretty big deal.” (21104)  “. . . We have to see the ophthalmologist, because he couldn't see in one eye, and they were like, “oh the graft-versus-host disease is in his eye,” which is causing all these issues of his vision being so blurry it. So, they gave us one prescriptions of eye drops. He had to use those every hour. It’s something that gets costly, because it's over the counter, but it's what he needs for his vision.” (21201)  “We had to stay six hours away from home so we had to rent an apartment at the beginning. At first, we had the money in our savings so it was not a challenge. We used the money. It became a burden having to pay a mortgage, bills to pay at home. We had other kids back home that we had to buy groceries for, send to school, find somebody to care for them . . . We ended up using credit cards . . . So, we became more in debt. We lost our savings . . .” (21203) | Emerging adults may be in a position to assist caregivers with costs. |
| Caregivers shield AYAs from financial burden | - Fear AYAs will be “overwhelmed,” “worried, “anxious,” or “crushed.” - Hiding medical bills - Omitting information about the caregiver’s financial situation - Minimizing depth/specificity of financial information disclosed - Admitting financial challenges but assuring AYAs | “. . . it's not her responsibility. And I don't want to worry her. I just want her to focus on getting better . . .” (21107)  “When she was in the anxiety I made sure I hid it [bills] from her. 'Cause she was opening the mail a lot because of cards and things and sometimes our paperwork is just lying on the kitchen counter, so I made sure for her not to see it.” (21105)  “She was very aware [of financial challenges] . . . we don't burden her, but we talk about it . . . we've told her that if something ever comes back, if we ever have to do anything else medically, that we'll do it. We'll figure it out. We will *all* figure it out.” (11203) | Caregivers of adolescents more consistently and forcefully asserted it was their sole responsibility to think about and manage cancer-related expenses*.* |
| Caregivers seek and/or receive financial support from social networks and health care institutions/organizations | - Pursuing support from organizations like Make-A-Wish, - Pursuing support from family and friends - Pursuing support from community (school, employers, church) event-based, in-person, and social media fundraisers   Pursuing support from insurance companies, hospitals, and assigned case workers and navigators | “. . . sometimes our family members, like my husband's parents, will ask and make sure that we're okay financially, and we say we're fine. I know that they would help out, but we couldn't ask them unless we were truly desperate.” (11102)  “Through his schools they were selling bracelets and they bring him, I think it was like a thousand dollar gift. That was around Christmas. There are some groups there, he used to play football, so the football team will bring dinner one day and stuff like that.” (21112)  “I just feel that if there's stuff out there, have it available for people to know . . . Someone ended up giving this book of . . . the burden of the financial aspect of what you're going to endure going through it. By that time, we had already been going through it for like a year.” (21201)  “Make it easier for people to know that they can get some type of assistance through the social services programs right away. I think we had to wait. We couldn't go right away. We had to wait until her bills accumulated to a certain amount of money. That, I hated.” (11208) |  |
| Caregivers desire help navigating the healthcare system and finding resources | - Receiving information about procedures, costs, and internal/external resources too late or in fragments. - Desiring timely face-to-face meetings with one dedicated financial resource officer or navigator - Desiring clear written documentation of costs and resource contact information is provided in addition to email summaries   Desiring assistance managing financial deadlines | “Like I said, maybe just during the initial sit down to kind of ... Even if it was just asked or told, ‘Hey if you have some questions or you need help to walk you through this for any kind of reason, then ‘Here's who . . . This is who you would talk to,’ or, ‘Here's where you would go to get that support if you need it.’ I just don't recall that happening.” (21102)  “I think that they could like tell us everything that they help with here. 'Cause it's like it comes in bits and pieces. (21107)  “There needs to be some kind of service, like some kind of financial, something like they would take over making sure your bills are paid, when you got so much going on you forget stuff. First it was like, you stayed in the hospital five days every two weeks.” (21111) |  |
| Caregivers use strategies to prepare for and adapt to cancer-related costs | - Delaying unnecessary travel and vacations - Reducing non-essential expenses for themselves and their children - Maximizing financial resources (e.g., applying for loans, tapping into savings, working longer hours) - Researching treatments, hospital procedures, and insurance systems to plan ahead financially - Delaying their own medical care - Delaying filling AYA prescriptions - Reevaluating spending philosophy - Saving (more) money for the future | “So he had money saved for college and whatever, and we had to sort of dip into that because we didn't have enough savings.” (21207)  “Well, I've made some decisions - right before <child>'s diagnosis, I had my right cataract, I had a cataract taken out of my right eye. After <child>'s diagnosis, first I put it off because of the diagnosis and I just now have gotten the money together. I think I'm gonna be able to have that done next month. Without it being a money issue, I would have never waited this long. It has been terrible, but I think I have the money now to get the left one done.” (21104)  “I immediately contacted my insurance company and I wanted to know all the ins and outs - the caps, what they would cover.” (21102)  “Helping my older daughter with some of her wants. She has everything she needs at school, but there have been some things she wanted to do or participate in that we haven't been able to do. There have been some things that <child> might have wanted to do at school that we haven't been able to do. Things like not buying anything above the minimum for her graduation things, the cap and gown and that sort of thing, just doing the absolute minimum of that sort of thing.” (21105) | Caregivers of adolescents often reported the need to adjust spending that impacted other young children in the family. |
| Caregivers worry about AYAs’ uncertain medical and financial future | - Worrying about uncertain cancer treatment options, duration, and insurance coverage - Worrying about future cancer-related sequelae or other cancer/ non-cancer-related health problems - Worrying about AYAs ability to pay for future medical expenses | “. . . we've only received statements through May, so we're not really sure what's going to come yet, after. We haven't received those in the mail yet . . . after May we aren't really sure what's going to come. And we don't know where this is going to turn. We know we're here and they've been great to us here and our insurance is good here, but you know what if something happens and that all changes?” (11201)  “Right now, I think the health insurance is not allowed to hold pre-existing conditions against people and that's a good thing. If that changes, I really don't know where that would leave her. I do worry that she might only qualify for extremely high deductible plans or something, but there's nothing we can do about that, so I haven't looked too far into that, but she's gonna need - there are different tests she's gonna have to have periodically, regardless of whether there are any symptoms or you're looking for cancer reoccurrence, but based on the chemo, like she has to have an echocardiograms frequently and that sort of thing and that's gonna go on forever.” (21104) | Caregivers of adolescents worry about patients’ future because adolescents often had not begun to make career decisions.  Caregivers of adolescents worry cancer may interfere with developmental life milestones that will impact their future financial health. |
