# Supplementary material for: Financial burden for caregivers of adolescents and young adults with cancer
Source: Psychooncology. Author manuscript; Available in PMC 2023 Aug 1. (PMC9540021; doi:10.1002/pon.5937)
Supplement: Supplement 3 - Financialburdenforcaregiversofadolescentsandyoungadultswithcancer [file NIHMS1831818-supplement-Supplement_3_-_Financialburdenforcaregiversofadolescentsandyoungadultswithcancer.docx]

**Supplementary Table 4.** COVID-19-related Financial Impacts and Exemplar Quotes

| **COVID-19-Related Impact** | **Exemplar Quote** |
| --- | --- |
| Job loss or reduction in work hours | “My wife's in sales and she's on the road. Besides the fact that she's going to the doctor's appointments, for a month and a half to two months, she couldn't go out. Which means she couldn't visit clients, which ultimately has affected income-wise. I build houses for a living. For a month and a half, we didn't talk to anybody. I mean, same thing. Profitability of my workplace has gone down, which ultimately will affect me.” (11206) |
| Uncertain nature of the pandemic and its long-term impact on the job market | “I think it's just the unknown. Furniture (industry in which her husband works) is not exactly essential, so that was always in my mind . . . for the future.” (11102) |
| Increase in household size | “. . . having extra people back in the house that weren't really budgeted to feed. So just expenses related to having more people in the house . . . just having extra people to feed and larger electric bills . . .” (21202) |
| Cost of goods and services increase | “Things have gotten more expensive. I know they said they weren't supposed to get more expensive, but groceries have gotten more expensive. Everything has gotten more expensive . . .” (11205) |
| Cost of purchasing pandemic-related products, masks, and cleaning supplies | “ . . .with COVID, having to make sure we have face covering. And for her, not a cloth mask, but actual mask. I had to purchase some and I probably wouldn't have purchased as many, but I just wanted to make sure she was safe whenever we went out. And, of course, they upped the price on these things, now, with everything going on.” (21202) |
| Reduced home-based caregiving and support | “We would have more family support (before COVID-19). We can't have any visitors or anything so that has been a big one. When his treatments started, we had a big support of people, of our family. We haven't had any contact or anything with the family since March because of COVID.” (21204) |
| Finding or affording medications due to limited inventory | “There were a couple of times that couldn't get her medication, because they didn't have any. It was out of stock. I had to go to several different pharmacies and get the hospital involved if they could find her medication because the pharmacy was out . . . and name brand was too expensive . . . I'm thinking people were panicking . . .” (21203) |
